# ^99m^Tc-DTPA Renal Dynamic Imaging Method May Be Unsuitable To Be Used as the Reference Method in Investigating the Validity of CDK-EPI Equation for Determining Glomerular Filtration Rate

**DOI:** 10.1371/journal.pone.0062328

**Published:** 2013-05-01

**Authors:** Peng Xie, Jian-Min Huang, Xiao-Mei Liu, Wei-Jie Wu, Li-Ping Pan, Hai-Ying Lin

**Affiliations:** 1 Department of Nuclear Medicine, The Third Hospital, Hebei Medical University, Shijiazhuang, P.R. China; 2 Department of Nephrology, The Third Hospital, Hebei Medical University, Shijiazhuang, P.R. China; University of São Paulo School of Medicine, Brazil

## Abstract

**Objective:**

To compare the measurements of glomerular filtration rate (GFR) determined by ^99m^Tc-diethylene triamine pentaacetic acid (^99m^Tc-DTPA) renal dynamic imaging with those estimated by Chronic Kidney Disease Epidemiology Collaboration (CDK-EPI) equation and to identify a more accurate measurement of GFR of chronic kidney disease (CKD) patients in clinical practice.

**Methods:**

The GFR was determined simultaneously by 3 methods: (a) dual plasma sample clearance method (tGFR); (b) renal dynamic imaging method (dGFR); (c) CDK-EPI equation (eGFR). The tGFR was employed as the reference method. The correlation, regression, and limit of agreement of dGFR and eGFR were used to demonstrate the validity of the two methods. The comparison of bias, precision, and accuracy between dGFR and eGFR was analyzed to identify the most suitable method. The analysis of bias, precision and accuracy was repeated after stratifying patients by a measured tGFR cutpoint of 60 ml·min^−1^·(1.73 m^2^)^−1^.

**Results:**

A total of 149 patients were enrolled. Both dGFR and eGFR correlated well with tGFR and the regression equation of dGFR and eGFR against tGFR was respectively Y = −4.289+0.962X (r = 0.919; RMSE = 14.323 ml.min^−1^. (1.73 m^2^)^−1^; *P*<0.001) and Y = 2.462+0.914X (r = 0.909; RMSE = 15.123 ml.min^−1^. (1.73 m^2^)^−1^; *P*<0.001). In addition, Bland-Altman analysis showed preferable agreement between the two methods and the reference method. The comparison revealed that eGFR, compared with dGFR, showed better performance on bias and 50% accuracy and similar performance on other indexes in the whole cohort and the lower-GFR subgroup, whereas in the higher-GFR subgroup the difference of the two methods was not significant in all parameters.

**Conclusions:**

Although both CDK-EPI equation and renal dynamic imaging can be used to determine the GFR of CKD patients, CDK-EPI equation is more accurate than renal dynamic imaging. As a result, ^99m^Tc-DTPA renal dynamic imaging may be unsuitable to be used as the reference method in investigating the validity of CDK-EPI equation.

## Introduction

Chronic kidney disease (CKD) can lead to serious complications and even death, and the prevalence of CKD is so high that it has been a worldwide epidemic and public health problem.[1]–[4] Glomerular filtration rate (GFR), the best overall index of renal function in health and disease, can be evaluated by several approaches. Inulin clearance, the gold standard for GFR determination, can not be applied widely in clinical practice because of its technical complexity and time-consuming procedure. Thus, many methods are developed to estimate GFR in order to obtain more accurate value and simpler procedure, including the equations based on serum creatinine and on serum cystatin C, and renal dynamic imaging method. [5]–[10] However, none of these methods can be extremely accurate to calculate GFR of CKD patients, so the discussion about which one is more appropriate is continuing.

Gates developed a simple method to determine GFR named Gate's method in 1982[10] and modified it in 1983[11], and because GFR can be calculated simultaneously by available computer system when the patient is undergoing renal dynamic imaging, this method can also be named renal dynamic imaging method. It has been the most commonly used method to evaluate GFR due to the following advantages: unilateral renal blood flow and kidney function can be obtained; more information is involved in the timed uptake curve (e.g. unilateral small renogram is helpful to the diagnosis of renovascular hypertension); the whole procedure takes approximately 20 min only; the collection of blood and urine can be avoided; and its result will not be interfered by the diet of the patient.

Recently, a new serum creatinine-based equation was reported by the Chronic Kidney Disease Epidemiology Collaboration (CDK-EPI), which was developed based on a large data set pooled from research and clinical populations, including renal disease patients and healthy individuals, in order to provide a more accurate method for estimating GFR, especially in the normal and higher ranges of GFR.[7] The validity of this equation was widely investigated and the results indicated that CDK-EPI equation performed well with less bias and greater accuracy than the Modification of Diet in Renal Disease equation.[12]–[18]

Although the renal dynamic imaging method has been employed as the gold standard in a few studies about assessing the performance of CDK-EPI equation[14], [15], [19], [20], [21], little is known about whether renal dynamic imaging method is more accurate than CDK-EPI equation in determining GFR. So we believe it's necessary to compare the two approaches mentioned above and the objective of the present study is to validate the applicability of the two methods and to provide some suggestions about whether it is reasonable to use renal dynamic imaging method as the gold standard to evaluate the validity of CDK-EPI equation.

## Subjects and Methods

### Ethics Statement

The study was approved by Hebei Medical University ethical committee on human experimentation and performed in accordance with the Helsinki Declaration, and all the participants signed informed consent.

### Patients

The diagnostic standard for CKD was achieved according to the National Kidney Foundation-Kidney Disease Outcomes Quality Initiative (K/DOQI) clinical practice guidelines, [22] however, in this study the diagnosis of CKD did not depend on the GFR but rather on kidney damage irrespective of the level of the GFR. The patients with CKD aged 18 years or older were enrolled. The patients who met the following criteria were excluded: with complications related to acute kidney function deterioration; with therapy of renal replacement; with edema, cardiac insufficiency, pleural effusion or abdomen effusion; with disabled limb; with treatment of cimetidine or trimethoprim that could affect the concentration of serum creatinine.[23]–[25] The patients were categorized into 2 subgroups based on the GFR calculated by reference method: lower-GFR subgroup (tGFR<60 ml·min-1·(1.73m2)-1) and higher-GFR subgroup (tGFR>60 ml·min-1·(1.73m2)-1).

### GFR measurement by dual plasma sample clearance method (tGFR)

True GFR (tGFR) was measured by using dual plasma sampling method and renal dynamic imaging was implemented simultaneously. Heparin anti-coagulated blood samples were taken 2 and 4 h after administrating 99mTc-diethylene triamine pentaacetic acid (99mTc-DTPA) from the opposite forearm. Plasma was separated (3 ml anti-coagulated blood centrifuged for 15 min at a speed of 1500 g), and radioactivity in the plasma (1 ml) was counted in multi-function well counter (CRC-25R multi-function instrument from CAPINTEC.INC,USA). The GFR was estimated from a single exponential formula derived from the blood samples between 2 and 4 h after injection. [26]

### GFR measurement by renal dynamic imaging method (dGFR)

99mTc-DTPA was prepared 30∼60 min prior to injection using a current DTPA kit (SHIHONG Pharmaceutical Center, Beijing, P.R. China) and instant thin layer chromatography was performed on all DTPA preparations to confirm the labeling efficiency >98%. The patients were hydrated with 500 ml water prior to examination. The patients lay down on the bed, and then a 6 s count of the syringe containing 99mTc-DTPA was performed before a bonus of intravenous injection of 175 MBq 99mTc-DTPA was administered to them. A SPECT (Infinia, General Electric Co, USA) was employed to complete the examination equipped with a low-energy general-purpose parallel-hole collimator, window width of 20%, 140 kev energy peak and frames of 128×128 matrix. The post-injection syringe was counted similar to pre-injection. The region of interest (ROI) was drawn manually on the frame of kidney and the semi-lunar background was placed around the lower, outer renal margin. After the patient's data about weight and height were input into an online computer, the dGFR was automatically calculated by commercially available software according to the Gate's algorithm.

### GFR measurement by CDK-EPI equation (eGFR)

The serum creatinine was measured in the same day when the rGFR and dGFR was administrated. The normal range of Scr was 58∼110 µmol/L with enzymatic method. All the tests were measured on the same automatic biochemical analyzer (VITROS 5.1, Johnson Company,USA) in a single laboratory, and the reagents were served by the same company which provided the machine. The CDK-EPI equation is as follows [7]:

Female with the concentration of serum creatinine ≤62 µmol/L,

eGFR = 144×(Scr/0.7)−0.329×(0.993)age;

Female with the concentration of serum creatinine >62 µmol/L,

eGFR = 144×(Scr/0.7)−1.209×(0.993)age;

Male with the concentration of serum creatinine ≤80 µmol/L,

eGFR = 141×(Scr/0.9)−0.411×(0.993)age;

Male with the concentration of serum creatinine >80 µmol/L,

eGFR = 141×(Scr/0.9)−1.209×(0.993)age

The unit of Scr and age is mg/dL and year respectively.

### The Normalization of GFR

The GFR (ml/min) obtained by the three approaches was normalized for a body surface area of 1.73 m^2^ according to Haycock's equation [27].

### Statistical Analysis

Quantitative variables were presented as mean±SD or median, and the comparison of quantitative variables was assessed by T-test or Wilcoxon Test. The diagnostic performance of dGFR and eGFR was determined by calculating linear correlation and regression, bias (defined as dGFR minus tGFR or eGFR minus tGFR), precision (SD of bias) and accuracy (proportion of GFR estimates within 15%, 30% and 50% deviation of tGFR). Additionally, Bland-Altman method was employed to evaluate the agreement of the two methods. Paired samples' t-test was used to compare the bias, and F-test was performed to compare the precision [28]. The accuracy of dGFR and eGFR was compared by employing McNemar test. A value of P<0.05 was considered statistically significant. Statistical analysis was made with SPSS (version 11.0, SPSS. Chicago IL, USA), SAS 8.0 (version 8.0, SAS Institute Inc, Cary, NC) and Medcalc for Windows 4.3 (version 4.3, Medcalc software, Mariekerke, Belgium).

## Results

The study included 73 males and 76 females with a mean age of 53.33±16.89 and 57.63±14.68 years respectively and there was no significant difference in the age of the two genders (t = −1.575, P = 0.117). They were given a wide variety of clinical diagnoses including chronic glomerulonephritis in 50 cases, diabetic nephropathy in 35 cases, chronic pyelonephritis in 27 cases, hypertensive nephropathy in 11 cases, chronic interstitial nephritis in 7 cases, IGA nephropathy in 6 cases, polycystic kidney disease in 4 cases, and other causes or unknown causes in the remaining 9 cases. The value of serum creatinine ranged from 69.00 µmol/L to 1482.8 µmol/L with mean 190.31 µmol/L and SD 182.18 µmol/L.

**Figure 1:**
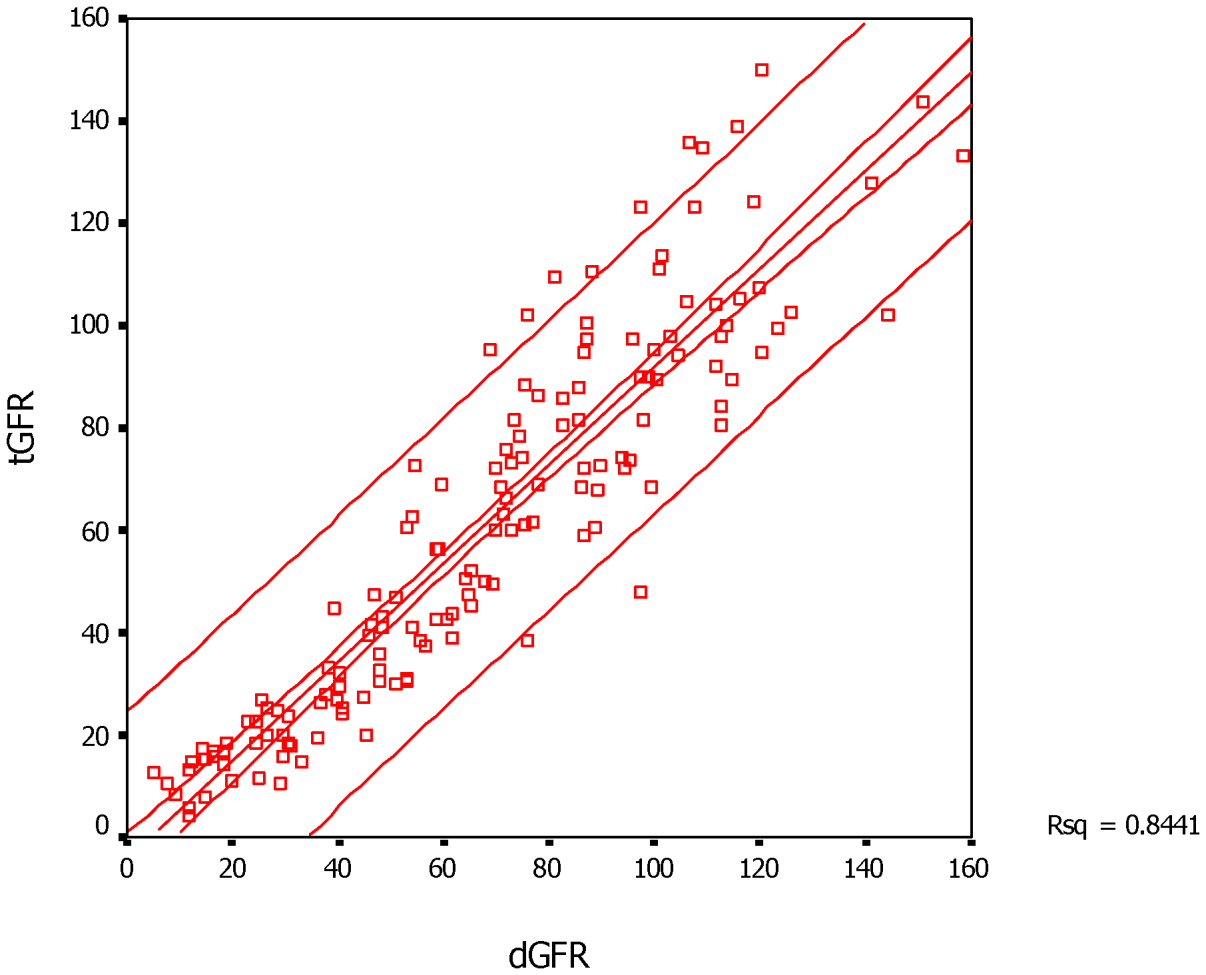
The scatter plot and linear regression of tGFR versus dGFR in 149 subjects. The center line represents the regression line and the surrounding lines show the 95% confidence interval of tGFR for a given dGFR. Abbreviations: dGFR- the GFR estimated by dynamic renal imaging method, tGFR- the GFR measured by dual plasma sampling method.

**Figure 2:**
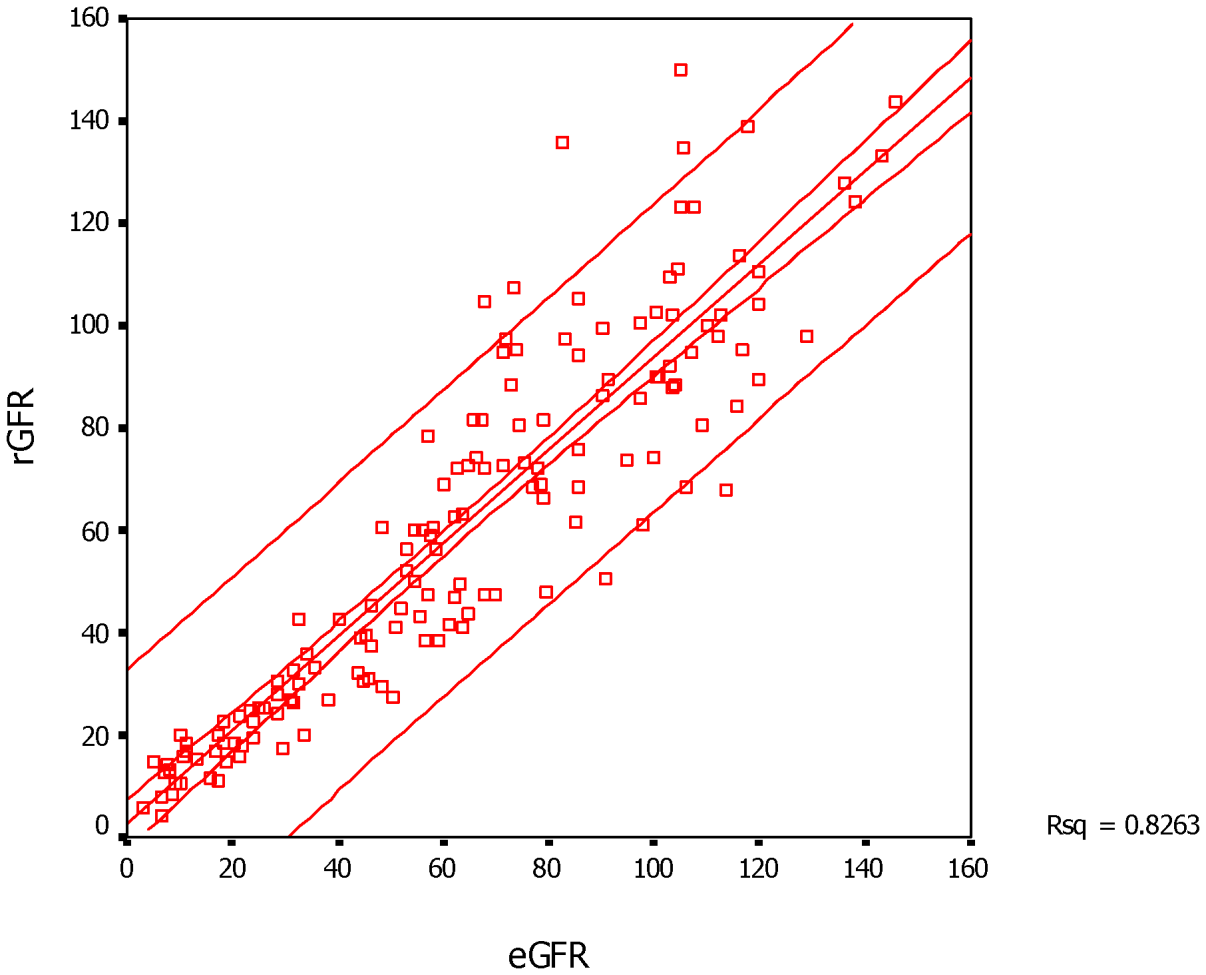
The scatter plot and linear regression of tGFR versus eGFR in 149 subjects. The center line represents the regression line and the surrounding lines show the 95% confidence interval of tGFR for a given eGFR. Abbreviations: eGFR- the GFR estimated by CDK-EPI equation method, tGFR- the GFR measured by dual plasma sampling method.

**Figure 3:**
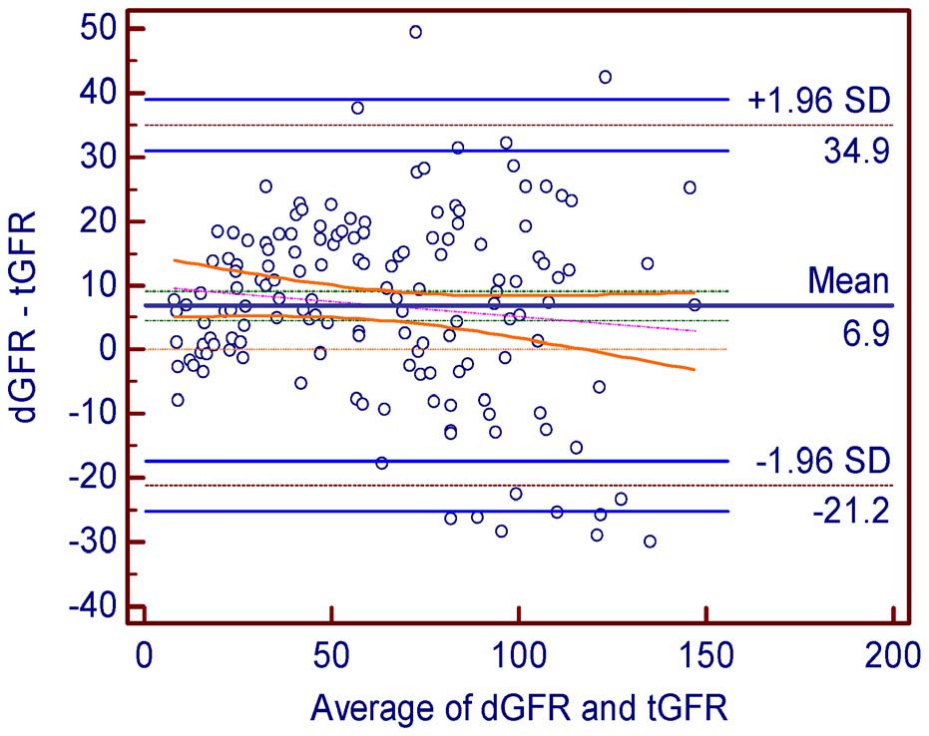
Bland-Altman plot showing the disagreement between dGFR or eGFR and tGFR. The solid long line indicates the mean of difference and the dotted long line represents the 95% limits of agreement. Abbreviations: dGFR- the GFR estimated by dynamic renal imaging method, eGFR- the GFR estimated by CDK-EPI equation method, tGFR- the GFR measured by dual plasma sampling method.

**Figure 4:**
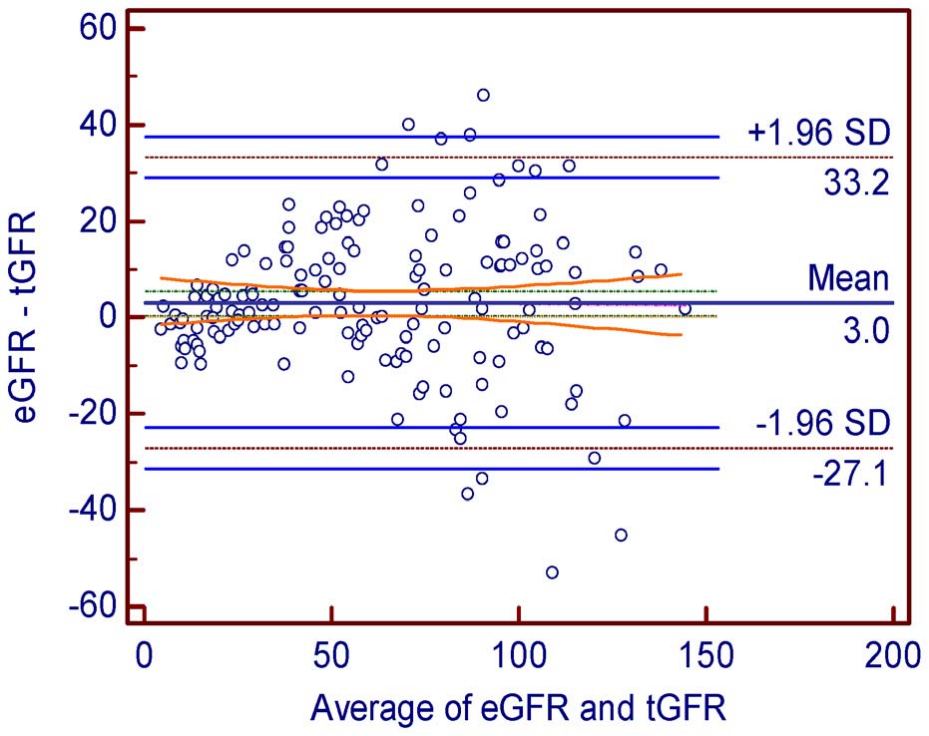
Bland-Altman plot showing the disagreement between dGFR or eGFR and tGFR. The solid long line indicates the mean of difference and the dotted long line represents the 95% limits of agreement. Abbreviations: eGFR- the GFR estimated by CDK-EPI equation method, tGFR- the GFR measured by dual plasma sampling method.

The GFR measured by dual plasma sample clearance method (tGFR), renal dynamic imaging method and CDK-EPI equation (eGFR) was 60.37±36.16, 67.22±34.54, and 63.38±35.97 ml·min-1·(1.73m2)-1, respectively, and eGFR showed similar value with tGFR (Z = −0.802, P = 0.423), but the value of dGFR was higher than that of tGFR (t = −2.390, P = 0.018). Both renal dynamic imaging method and CDK-EPI equation correlated well with dual plasma sample clearance method, and the coefficient correlation was 0.919 (P<0.001) and 0.909 (P<0.001), respectively, with no significant difference (Z = 0.520, P = 0.603). Furthermore, the regression equation of dGFR and eGFR against tGFR was Y = −4.289+0.962X (r2 = 0.844; RMSE = 14.323 ml·min-1·(1.73m2)-1, P<0.001) and Y = 2.462+0.914X (r2 = 0.826; RMSE = 15.123 ml·min-1·(1.73m2)-1, P<0.001), respectively (Fig. 1 Fig. 2). Bland-Altman analysis showed preferable agreement between the two methods and the reference method and the 95% limit of agreement for dGFR and eGFR was 56.21 ml·min-1·(1.73m2)-1and 60.31 ml·min-1·(1.73m2)-1 (Fig. 3 Fig. 4) respectively.

**Figure 5:**
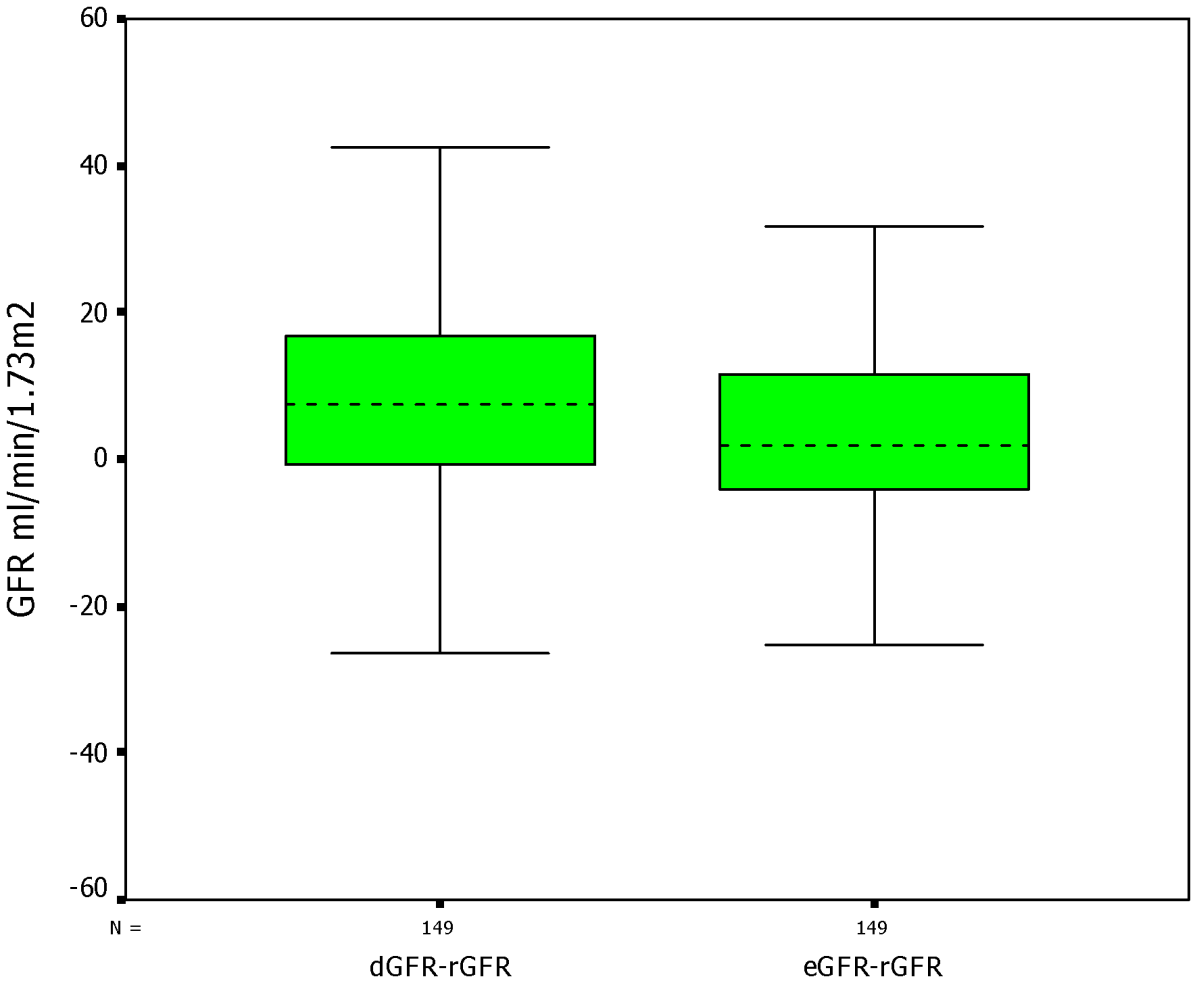
Boxplots of difference in GFRs. The dotted lines indicate the median and the continuous lines represent the quartiles values of difference. Abbreviations: dGFR- the GFR estimated by dynamic renal imaging method, eGFR- the GFR estimated by CDK-EPI equation method, tGFR- the GFR measured by dual plasma sampling method.

In the whole cohort, the mean bias of dGFR and eGFR was 6.85 ml·min-1·(1.73m2)-1 and 3.01 ml·min-1·(1.73m2)-1 (Fig. 5) respectively, and the comparison showed significant difference in the performance of bias (t = 3.191, P = 0.002). Although the percentage of eGFRs within 30% and 15% of tGFR was not higher than that of dGFR (71.14% vs 66.44% for 30% accuracy, P = 0.419; 48.99% vs 41.61% for 15% accuracy, P = 0.207), eGFR made some improvement in 50% accuracy over dGFR (91.28% vs 83.22%; P = 0.017). The precision of eGFR was not significantly lower than that of dGFR (14.34 ml·min-1·(1.73m2)-1 vs15.39 ml·min-1·(1.73m2)-1; F = 1.152, P = 0.831). The performance of bias, precision and accuracy in the lower-GFR subgroup was almost completely consistent with that in the whole cohort, whereas the difference of those indexes in the higher-GFR subgroup did not reach significance (Table.1).

**Table 1:** The comparison of the dynamic renal imaging method and the CDK-EPI equation on the performance in estimating GFR.

Method	Bias (Mean)	Precision (SD)	Accuracy with 50%, %	Accuracy with 30%, %	Accuracy wwith 15%, %
Whole cohort (n = 149)					
dGFR	6.85	14.34	83.22	66.44	41.61
eGFR	3.01^**^	15.39*	91.28^**^	71.14*	48.99*
GFR<60 (n = 76)					
dGFR	10.40	9.86	67.11	43.42	27.63
eGFR	5.04^**^	9.95*	86.84^**^	57.89*	39.47*
GFR>60 (n = 73)					
dGFR	3.15	17.14	100	90.41	56.16
eGFR	0.90*	19.36*	95.89*	84.93*	58.90*

* P>0.05 comparing the bias, precision and accuracy of dGFR with those of eGFR. ^**^P<0.05 comparing the bias, precision and accuracy of dGFR with those of eGFR.

Abbreviations: dGFR- the GFR estimated by dynamic renal imaging method, eGFR- the GFR estimated by CDK-EPI equation method.

## Discussion

The CDK-EPI equation, developed in 2009, has been proved to be suitable to determine the GFR in Japanese, American, Australia, Spain, and Chinese populations [14], [16]–[18], [29]. Like previous researches, the satisfactory coefficient correlation (r = 0.909) and no significant difference (P = 0.423) against the reference method in our study proved that CDK-EPI equation can be applied as an ideal formula to estimate the GFR of Chinese patients with CKD. However, some studies used renal dynamic imaging as the reference method in investigating the application of this equation [14], [15], [19], [20], [21].

Because of the satisfactory accuracy and relative simplicity, 99mTc-DTPA dual plasma sample clearance method, recommended as the reference approach in determining GFR by the Nephrology Committee of Society of Nuclear Medicine [26], was widely used as the gold standard in clinical trials, and it was employed as the true means to calculate the GFR in our study. In the comparison between CDK-EPI equation and renal dynamic imaging method in our study, the bias and 50% accuracy of the former showed better performance than that of the latter in the whole cohort and lower-GFR subgroup, potentially revealing that the renal dynamic imaging method was less accurate than CDK-EPI equation. Nonetheless, the two methods performed similar capability in determining GFR in the higher-GFR subgroup.

The previous researches showed that renal dynamic imaging method could overestimate the true GFR [30], and our study also proved that the GFR measured by this method was higher than that by reference standard (P = 0.018). Although a large number of factors could influence the accuracy of renal dynamic imaging method including the sketching of region of interesting [31], the dosage of administration [32] etc., the leading one, in our opinion, was that the empirical equation, creatinine clearance method, was employed as the reference method when Gates developed renal dynamic imaging method. Because creatinine clearance method was not as accurate as inulin clearance, therefore, the precision of renal dynamic imaging method might be elevated by choosing more accurate approach as reference standard instead of the creatinine clearance method.

The limitations of the present study include the following ones. First, the method of determining the concentration of creatinine in our study, the enzymic assay, is different from the Jaffe method used by the CDK-EPI researchers who developed the equation, [7] but the enzymic assay obviates the poor specificity of the Jaffe method [33]. Moreover, the sample size was small so we did not compare the validity of the two methods according to the stages and causes of CKD and the ages of patients.

In conclusion, although both CDK-EPI equation and renal dynamic imaging method can be used in determining glomerular filtration rate of chronic kidney disease patients in clinical practice, 99mTc-DTPA renal dynamic imaging method is less accurate than CDK-EPI equation, therefore, it is maybe unsuitable to be used as the reference method in investigating the validity of CDK-EPI equation.
